# Self-reported incidents of violence towards nurses working in acute psychiatric units

**DOI:** 10.4102/curationis.v46i1.2350

**Published:** 2023-06-29

**Authors:** Ntombiyakhe Bekelepi, Penelope Martin

**Affiliations:** 1 School of Nursing, Faculty of Community Health Science, University of the Western Cape, Cape Town, South Africa

**Keywords:** acute psychiatric unit, nurses, physical violence, verbal abuse, violent incidents

## Abstract

**Background:**

Acute psychiatric units are found to be stressful working environments because of the nature of illness patients present with.

**Objectives:**

This study aimed to determine self-reported incidents of physical and verbal violence towards nurses working in acute psychiatric units in Western Cape, South Africa.

**Method:**

A questionnaire was used to collect data. Chi-square test was performed to determine association between gender, category and experience of violence. Mann–Whitney *U* test was carried out to determine associations between years of employment and the likelihood of experiencing physical violence and verbal abuse.

**Results:**

Overall physical violence 35 (34.3%) and verbal abuse 83 (83%) incidents. Most female respondents reported both physical violence (74.2%, *n* = 26) and verbal abuse (72.2%, *n* = 60), with (56.2%, *n* = 18) professional nurses reporting physical violence. Years of employment was statistically significantly associated with the likelihood of nurses experiencing physical violence (*p* = 0.007).

**Conclusion:**

Most respondents (74.2%, *n* = 26) were females and they mostly experienced physical violence and verbal abuse while 28.2% (*n* = 29) were males. Years of service were associated with the likelihood of experiencing physical violence.

**Contribution:**

The knowledge gained will add on existing knowledge about the challenge of violence experienced by nurses in the workplace and might have an influence on policymakers.

## Introduction

Globally, workplace violence in healthcare is a major public health concern. The prevalence of workplace violence towards nurses is significantly high in psychiatry settings compared with all other healthcare settings (Dean, Butler & Cuddigan 2021; Odes et al. 2021). On average, nurses are three times more likely to experience violence in the workplace compared with their counterparts (Liu et al. 2019; WHO 2010). They have close contact with patients and their families, which results in numerous interactions that can place them at risk of being exposed to violent incidents (Niu et al. 2019).

Patients are admitted in acute psychiatric wards in order to manage their acute psychotic symptoms. These symptoms may include but are not limited to hallucinations, delusions, and a lack of insight into their mental illness. These patients often display violent behaviours towards nursing staff. A review conducted by Whiting, Litchtenstein and Fazel (2021) reaffirmed that the risk of violence in psychiatric settings is higher compared with other settings. Boafo, Hancock and Gringart (2016) reported that most incidents of violence against nurses are not reported because of no action being taken to investigate or no action taken against the perpetrator of violence. Studies also show that nurses working in psychiatric setting perceive violence as part of their working environment (Agbornu, Boafo & Ofei 2022; Moylan et al. 2016).

The WHO (2012) reported the incidence of workplace violence towards healthcare workers as ranging between 8% and 38%, with more nurses encountering violence annually especially those working in psychiatric environment (Renwick et al. 2019). A study conducted in Brazil by Bernardes et al. (2020) showed that violence is a common feature of the workplace for nurses with verbal abuse being the most common type of violence, leading to stress and dissatisfaction with their work. Workplace violence against nurses has been reported in most regions globally in the last decade, in countries such as in Malawi (Banda, Mayers & Duma 2016); China (Jiao et al. 2015); Italy (Ramacciati, Ceccagnoli & Addey 2015); United States (Gates, Gillespie & Succop 2011); Germany (Franz et al. 2010); Taiwan (Shiao et al. 2010); Egypt (Abbas et al. 2010) and South Africa (Kajee-Adams & Khalil 2010). In a study carried out in Saudi Arabia, Al-Otaibi, Gamal and Edesouky (2016) reveal that nurses working in psychiatric settings had the highest rate of exposure to violence (84%), with psychiatric nurses working in acute care units are at an increased risk and are frequently subjected to patient violence (Hiebert et al. 2021). Patients admitted in this unique type of secure environment are often involuntary committed because of the severity of their mental illness and can be a danger to themselves or others (Da’seh & Obaid 2017). According to Sobekwa and Arunachallam (2015), nurses are therefore exposed to different challenges in their working environment. Staff have to use limit setting to control patient disruptive behaviour, which can lead to the risk of evoking negative feelings among patients (Salzmann-Erikson 2017). These limit setting include the locking of the acute psychiatric units, which limit patients from moving freely because of restrictions and disease characteristics (Niu et al. 2019). The unsafe work environment for mental health nurses not only impacts work stress but also reduces their life satisfaction (Itzhaki et al. 2015; Sobekwa & Arunachallam 2015).

Evidence shows that exposure to workplace violence has negative outcomes for nurses and organisations. Nurses experience negative effects such as increased stress levels, decreased work satisfaction and had adverse long-term health consequences following an exposure to violence (Friis et al., 2018; Itzhaki et al. 2018). The emotional effects of violence on nurses include but are not limited to psychological consequences with a high rate of stress and other sequelae such as depression, post-traumatic stress disorder (PTSD), and burnout (d’Ettorre & Pellicani 2017; Jalil et al. 2017; Stevenson et al. 2015). A study conducted by (Joubert & Bhagwan 2018) in the KwaZulu-Natal province of South Africa revealed that nurses in acute psychiatric wards experienced increased levels of burnout and frustration because of working under stressful situations. Findings further revealed that nurses experience complex challenges in acute psychiatric wards as they are always faced with aggressive and unpredictable patient behaviour. Work-related stress can have negative impact on healthcare providers’ professionalism, quality of care delivery, efficiency and overall quality of life (Sovodi et al. 2021). In another study conducted in the Gauteng province, South Africa by Nguluwe, Havenga and Sengane (2014), findings showed that the effects of violence on emotions of nurses working in acute psychiatric wards were negative feelings. In the long run, these nurses who have unresolved psychological and emotional trauma may present with symptoms of altered mental health (Nguluwe et al. 2014). Sobekwa and Arunachallam (2015) conducted a study in an acute ward in one of the psychiatric hospitals in Cape Town, participants reported shortage of staff as one of the challenges they face in acute wards as the staffing does not compliment patient ratio. This means nurses have to carry more workload on their shoulders. This has been observed by Sailaxmi and Lalitha (2015), that inadequate nurse patient ratio can contribute to stress in nurses. In their study, Mulaudzi et al. (2020), findings revealed that nurses were experiencing burnout because of work-related stress, which affected their passion for the profession. Nurses may develop attitude towards the patients, which compromises quality nursing care (Hylén et al. 2019). This was confirmed in a study by Hiebert et al. (2021) where participants reported that they are reluctant to work with patients with a history of violence; some were considering leaving their job altogether and with overall job dissatisfaction.

In South Africa, healthcare is administered by the Department of Health and has two parallel systems, which is public healthcare and private healthcare that operates in tandem with one another (Petersen et al. 2015). Mental healthcare in South Africa prior to 1997 was mainly institutionalised care and there was not much emphasis on curative therapy (Petersen et al. 2009). Following the shift with democratic dispensation in 1994, the concept of universal primary healthcare became centre of discussion in restructuring the healthcare system as stipulated in the White Paper for the transformation of health system (Department of Health 1997) and *National Health Care Act 61 of 2003*. A progressive *Mental Health Care Act 17 of 2002* was adopted and was seen as an important step towards addressing mental health as a public health issue (Lund et al. 2012). For the implementation of the *Mental Health Care Act 17 of 2002*, in 2013 a new Mental Health Policy Framework (MHPF) and Strategic Plan 2013–2020 were adopted, which are aligned to WHO Mental Health Action Plan that embraces task sharing and integration of mental health into primary healthcare services. Despite this milestone South Africa has achieved in having this policy, Marais and Petersen (2015) identified a weak health system and health system governance that make it difficult to implement the policy and deliver cost-effective interventions for scaled-up mental healthcare. A report by the South African Human Rights Commission (2017) highlighted that there is a prolonged and systemic neglect of mental health at the level of policy implementation in South Africa, which lead to a lack of resources. The report further states that South African government is under-investing in mental health, which has a major impact in distribution of resources.

There is a severe shortage of workforce in mental health both in low- and middle-income countries (Bruckner et al., 2011), with South Africa lacking specialised workforce needed to provide mental health services in the public sector and rural areas (Moodley, Wolvaardt & Grobler 2022). WHO (2017) reported that South Africa had 1.52 psychiatrists per 100 000 population compared with median number of psychiatrists per 100 000 populations of 2.1 for upper middle-income countries and 12.7 for high-income countries. There are also substantial disparities in the distribution of mental health workers between the private sector in South Africa as well as urban and rural areas. According to Sehularo (2016), despite the qualification in mental health nursing being in existence for 40 years, there is severe shortage of advanced mental nurse specialist in South Africa. This puts pressure on the mental health nurses who are prone to workplace violence.

## Problem statement

Despite numerous investigations aiming to understand, predict, and manage psychiatric inpatient violence against nurses, this still remains an occupational health challenge in South Africa and beyond. Even though much research on workplace violence has been carried out globally for decades in psychiatric hospitals and in South Africa in particular, there is a scanty information on the violence experienced by nurses working in acute psychiatric wards in psychiatric hospitals in the Western Cape; therefore, with this study, the researcher aims to bridge the gap with the knowledge that will be generated, which will help policymakers and management of these institutions in coming up with strategies that will assist in managing the situation. Given the need to identify and implement effective strategies to reduce psychiatric inpatient violence, this research aims to determine self-reported incidents of physical and verbal violence towards nurses in acute psychiatric units are needed.

## Objective of the study

To determine self-reported incidents of physical violence and verbal violence towards nurses in acute psychiatric units.

### Definitions of key concepts

#### Violence

The definition of violence is consistent with the WHO definition, which alludes to violence being the intentional use of physical force or power, threatened or actual, against oneself, another person or against a group or community that either results in or has high likelihood of resulting in injury, death or psychological harm (WHO 2002).

#### Verbal abuse

Verbal abuse was defined as any annoying or unpleasant act (words, attitude or actions) that creates a hostile work environment (Magnavita & Heponiemi 2012).

#### Physical violence

For the purpose of this study, physical violence refers to reported incidents of physical violence, and verbal abuse refers to reported incidents of verbal abuse by respondents.

Nurses included professional and non-professional nurses who are registered with the South African Nursing Council to practice nursing.

#### Non-professional nurse

An individual responsible for assisting professional nurses in the delivery of nursing care according to their scope of practice, under direct supervision of professional nurse (*Nursing Act 33 of 2005*). In this study, a non-professional nurse refers to nurses (enrolled nurse and nursing assistant) registered with the South African Nursing Council to obtain a practice licence in order to provide nursing care to mentally ill patients under supervision of professional nurses.

## Research method and design

A cross-sectional, quantitative survey design was conducted in acute units in three psychiatric hospitals in the Cape Town metropolitan area, Western Cape province, South Africa. This type of research design is appropriate if you intend to measure variables and to establish associations between variables. This design was most suited to describe incidents of physical violence and verbal abuse as reported by nurses working in acute care settings in the three psychiatric hospitals.

### Setting

The research study was conducted in six acute units of the three psychiatric hospitals in Western Cape province, South Africa. The Western Cape province is one of the nine provinces in South Africa, which is situated in the southern extremity of the African continent. It lies just to the south of greater interior plateau of Southern Africa. There are five districts in the province, with 32 district hospitals, six regional hospitals and three central hospitals, which provide tertiary care. There are four designated psychiatric hospitals in the Western Cape, of which only three agreed in taking part in the study. These hospitals provide mental health services for persons with acute psychiatric disorders and intellectual disabilities to the larger population of the Western Cape. The bed capacity of the three hospitals is 1504 in which 475 are dedicated to acute units, with total number of 856 nurses. The staff population that works in these acute units comprises of a multidisciplinary team of nurses, doctors, psychologist, occupational therapist and physiotherapist. The acute units are closed wards that admit patients who are mostly psychotic and have high risk of displaying violent behaviour. Patients who are admitted in this type of unique environment are usually involuntarily committed because of severity of their mental illness and can be dangerous to self and others.

### Study population and sampling strategy

The population of the study included all categories of nurses working in the three participating hospitals. After receiving ethical approval, the researcher visited all three hospitals and met with managers from the acute wards. Then managers introduced the researcher to the potential participants from both day and night shifts, where information about the study was shared with the nurses. An all-inclusive sampling was used and all categories of nurses working in the acute wards were recruited to participate in the study. The inclusion criteria required all nurses who had experienced violence while working in acute psychiatric wards, regardless of how long they had worked in the ward. Nurses who are involved directly in bedside caring of psychiatric patients were included in the study. All categories of nurses, that is, professional nurses and non-professional nurses (enrolled nursing assistants and enrolled nurses) were included. Those who were interested to take part in the study were given an envelope, which contained a consent form, an information sheet and a questionnaire.

### Data collection method

Data were collected using self-administered questionnaires. The questionnaire was adopted from a similar previous study by the World Health Organization (WHO et al. 2003). The 53-item, Likert-type Workplace Violence Survey Questionnaire, developed by the WHO, in conjunction with International Council of Nurses and Public Services International (2003) to survey experiences of physical and verbal abuse in the workplace over the last 12 months was used in this study (WHO et al. 2003). The questionnaire has been used in several studies across different countries such as Northern Taiwan (Niu et al. 2019), including South Africa (Steinman 2003). Permission was sought from WHO to adapt some sections of the questionnaire that were relevant to the study for the survey. The overall Cronbach’s alpha of the questionnaire was 0.85. The questionnaire was adapted and modified to the study needs. The questionnaire had four sections, that is, section A: demographic data, section B: report on physical workplace violence; section C: report on verbal abuse in the workplace and section D: participant’s opinions on workplace violence. Data were collected between March 2019 and May 2019.

A total number of 124 respondents received the self-report questionnaire along with written information about the study and consent forms. Of the 124 questionnaires, 114 (91.9%) were returned. After the exclusion of 11 questionnaires, which had missing data, 103 (83.1%) were analysed.

### Data analysis

The Statistical Package for Social Sciences (SPSS) version 25 was used for data entry and statistical analysis. Descriptive statistics for demographic data and physical violence and verbal abuse were calculated. Descriptive statistics were presented as a percentage and means. Frequency and percentages were used to quantify the type of violence, consequences for the reported perpetrator, and the response of the nurses who experienced violence. The Chi-square test and Mann–Whitney *U* test were used to assess association between demographic variables (gender, nursing category, experience) and physical violence and verbal abuse.

### Ethical considerations

Ethics approval was obtained from the Biomedical Research Ethics Committee, University of the Western Cape, Cape Town, South Africa (Reference: BM18/6/18), and the Western Cape Department of Health (Reference: WC_201809_005). Permission to conduct the study was obtained from the Chief Executive Officers of the selected psychiatric hospitals. Informed consent was obtained and voluntary participation in the study was explained. Participant information sheets were given to all participants which explained the aim, ethical considerations and guidelines for taking part in the study. Participants’ names were not linked to the data to ensure anonymity.

## Results

### Demographic information

The total number of respondents was 103, which yielded a response rate of 83.1%. The respondents’ ages ranged between 24 and 60 years with the mean age being 40.89 (s.d.: 10.36) years. Most of the respondents were female (*n* = 74, 71.8%) and 29 (28.2%) were males, more than half (*n* = 60, 61.22%) were professional nurses, and 38 (38.7%) were non-professional nurses (Table 1). The type of ward where respondents worked in was 100 (97.1%) in acute units. The mean number of years of employment was 12.83 years (s.d.: 10.725), and for years worked in a unit was 6.03 years (s.d.: 5.356) and total years as nurse was 15.99 years (s.d.: 12.207).

**TABLE 1:** Demographic data of respondents (*n* = 103).

Variables	Statistics			
	Mean	s.d.	*n*	%
**Age (mean in years) (*n* = 92)**	40.89	10.36	-	-
**Gender**				
Female	-	-	74	71.8
Male	-	-	29	28.2
**Nurse category (*n* = 98)**				
Professional nurses	-	-	60	61.2
Non-professional nurses	-	-	38	38.7
**Type of ward (*n* = 100)**				
Acute unit	-	-	100	97.1
**Experience**				
Years of employment (*n* = 99)	12.83	10.73	-	-
Years worked in unit (*n* = 93)	6.0	5.36	-	-
Total years as nurse (*n* = 100)	15.9	12.21	-	-

*Source:* Niu, S-F., Kuo, S-F., Tsa, H-T., Kao, C-C., Traynor, V. & Chou, K-R, 2019, ‘Prevalence of workplace violent episodes experienced by nurses in acute psychiatric settings’, *PLOS One* 14(1), e0211183. https://doi.org/10.1371/journal.pone.0211183

s.d., standard deviation.

### Frequencies of physical and verbal attacks for three hospitals

With regard to self-reported physical violence and verbal abuse within the last 12 months, the overall experience of reported physical violence was 35 (34.3%) and reported verbal abuse was 83 (80.6%). Of those participated in the study, 35 (34.4%) reported that physical violence was perpetrated by patients (Table 2).

**TABLE 2:** Frequencies of physical and verbal attacks.

Incidents	Type of violence			
	Physical violence		Verbal abuse	
	*n*	%	*n*	%
Yes	35	34.0	83	80.6
No	67	65.0	17	16. 5
Missing	1	1.0	3	2.9

**Total**	**103**	**100.0**	**103**	**100.0**

*Source*: Bernardes, M.L.G., Karino, M.E., Martins, J.T., Okubo, C.V.C., Galdino, M.J.Q. & Moreira, A.A.O., 2020, ‘Workplace violence among nursing professionals’, *Revista Brasileira De Medicina Do Trabalho* 18(3), 250–257. https://doi.org/10.47626/1679-4435-2020-531

### Factors associated with the incidents of physical and verbal violence

Of the total number (*n* = 103) of respondents, only 35 (34.0%) reported to have experienced physical violence, and 9 (25.7%) of those were males while the majority (*n* = 26) (74.2%) were females (Table 3). With nursing category, slightly more than half of respondents (*n* = 18, 56.2%) reported to have experienced physical violence were professional nurses, less than half (*n* = 14, 43.7%) who reported to have experienced physical violence were non-professional nurses. With the reported verbal abuse, 31 (51.6%) were professional nurses, while 47 (60.2%) were non-professional nurses. The Mann–Whitney *U* test showed a significant association between all categories of nurses and years of employment and the likelihood of experiencing physical violence (*p* = 0.007) (Table 3).

**TABLE 3:** Factors associated with the incidents of physical violence and verbal abuse.

Variables	Reported physical assault (*n* = 35)					Reported verbal abuse (*n* = 82)				
	Statistic		Test		*p*	Statistic		Test		*p*
	*n*	%	χ^2^	*U*		*n*	%	χ^2^	*U*	
**Gender**	-	-	0.081	-	0.776	-	-	0.394	-	0.530
Male	9	25.7	-	-	-	23	27.7	-	-	-
Female	26	74.2	-	-	-	60	72.2	-	-	-
**Category of staff**	-	-	0.636	-	0.425	-	-	0.633	-	0.426
Professional nurse	18	56.2	-	-	-	31	51.6	-	-	-
Non-professional nurse	14	43.7	-	-	-	47	60.2	-	-	-
**Experience (*n* = 34)**	-	-	-	2.707	0.007*	-	-	-	0.928	0.354
Total years as nurse	21.50	13.7	-	-	-	16.65	12.4	-		-
Years worked in unit	7.93	6.0	-	-	-	6.41	5.5	-		-
Years of employment	17.85	12.5	-	-	-	13.51	11.2	-		-

*Source:* Niu, S-F., Kuo, S-F., Tsa, H-T., Kao, C-C., Traynor, V. & Chou, K-R, 2019, ‘Prevalence of workplace violent episodes experienced by nurses in acute psychiatric settings’, *PLOS One* 14(1), e0211183. https://doi.org/10.1371/journal.pone.0211183

* , Significant at *p* < 0.05.

χ^2^, Chi-square; *U*, Mann-Whitney *U* test.

### Management of incidents

Most of the respondents (*n* = 81, 81%) reported that they were encouraged to report any incidents of violence to management or supervisor, and the encouragement was mostly done by hospital management (88.4%, *n* = 69). With regard to follow up on incidents of violence, 14 (45.1%) of the 31 (88.5 %) respondents, reported that incidents of physical violence were investigated, and 14 (93.3%) reported that those investigations were performed by management. Only 6 (26.0%) respondents reported that counselling was provided, and 16 (59.2%) reported that opportunity to speak about the incident was provided. With regard to consequences for the perpetrator, 11 (37.9%) reported that there was no action taken against the perpetrator of violence by hospital management. On a personal level, out of 35 (100%) respondents who had experienced physical violence, 30 (85.7%) reported the incident, but only 16 (45.7%) completed the incident form to report it, while 8 (23.4%) took some time off work following the physical violence incident. In terms of satisfaction with the way management handled incidents of physical violence, 10 (33.3%) respondents were satisfied while 11 (36.6%) were not satisfied.

With regard to management of verbal abuse incidents, 20 (26.3%) respondents reported that incidences were investigated. Of those 17 (89.4%) of respondents reported that incidents were investigated by management, 10 (20.4%) reported that counselling was provided, 26 (50.9%) reported that opportunity to talk about the incident was provided by the supervisors, 35 (61.4%), while (26.3%) reported that verbal warnings were issued to perpetrators.

In response to verbal abuse, 17 (21.4%) reported the incident, 8 (10.1%) completed incident form, while 16 (20.2%) reported no action taken against the perpetrator. In terms of satisfaction with handling of incidents by management, 22 (39.2%) reported that they were satisfied, while 18 (32.1%) were not satisfied (Table 4).

**TABLE 4:** Management of incidents (hospital and person).

Action	Physical violence		Verbal abuse	
	*n*	%	*n*	%
Incident investigated	14	45.1	20	26.3
Investigated by management	14	93.4	17	89.4
Opportunity to speak about incident	16	59.2	26	50.9
No action taken against attacker	11	37.9	35	61.4
Counselling provided	6	26.0	10	20.5
**Personal**				
Reported incident	30	85.7	17	21.5
Completed incident form	16	45.7	8	10.1
Satisfied with management of incident	10	33.0	22	39.2
Dissatisfied with management of incident	11	36.6	18	32.1

*Source:* Niu, S-F., Kuo, S-F., Tsa, H-T., Kao, C-C., Traynor, V. & Chou, K-R, 2019, ‘Prevalence of workplace violent episodes experienced by nurses in acute psychiatric settings’, *PLOS One* 14(1), e0211183. https://doi.org/10.1371/journal.pone.0211183

Note: Encouraged to report incidents (both physical and verbal: *n* - 81 (81.3%); encouraged by management (both physical and verbal): *n* = 69 (88.4%).

## Discussion

The aim of the study was to determine nurses’ self-reported incidents of physical and verbal violence. This study’s finding showed that verbal abuse was more prevalent than physical abuse (83%). The findings are similar to those of the study by Niu et al. (2019) where verbal abuse (78.8%) was reported more compared with physical violence. These findings are similar to the previous studies that reported that verbal abuse was the most common form of violence reported by nurses (Banda et al. 2016; Niu et al. 2019). This study showed that patients are the frequent perpetrators of both physical and verbal violence, which is consistent with findings by Kobayashi et al. (2020) where patients were identified as most frequent perpetrators of physical violence. Participants in this study expressed that they worry more about physical violence in their workplace, which is consistent with the study by Agbornu et al. (2022).

Findings revealed that of the respondents in the study, females were mostly indicated to have been both physically attacked (74.2%) and verbally abused (72.2%). These findings are similar to the study by Alyaemni and Alhudaithi (2016) who noticed that female nurses reported more verbal abuse than their male counterparts. In contrast, a study by Renwick et al. (2019) found that experience of physical violence was associated with being male and being longer in the nursing category. However, this study’s findings showed no statistically significance associated with gender and experiencing physical violence or verbal abuse, meaning that both male and female nurses had equal risk of exposure to violence perpetrated by patients.

This study’s findings allude to professional nurses being more likely to experience physical violence (56.2%) than non-professional nurses. This could be that professional nurses are being trained in handling aggressive patients and are therefore assigned to manage patients in acute psychiatric units. This finding is consistent with the findings of the study by Stevenson et al. (2015) in which professional nurses reported to have experienced verbal and physical violence. There were no statistically significant differences between the nursing category and experiencing physical violence and verbal abuse. The study’s findings showed statistically significant association with years of employment and likelihood of experiencing physical violence (*p* = 0.007). This finding is consistent with the study of Akanni et al. (2019), which revealed that there was an association in having longer years of experience as a nurse and experiencing physical violence. The more experience the nurse had, the more he or she was vulnerable to physical violence.

Management of incidents by hospital management and the person experiencing violence, this study’s findings showed majority of respondents reported that they were encouraged by management to report incidents. This is similar to a study by Niu et al. (2019) which revealed that majority of participants reported that they were encouraged to report violence in the workplace. In response to physical violence, respondents in this study reported that incidents of violence were investigated by management and this finding is similar to that of the study by Alyaemni and Alhudaithi (2016) in which participants reported that incidents were investigated. Respondents were provided with counselling and opportunity to speak about incidents, and this is similar to results from studies by Niu et al. (2019) and Alyaemni and Alhudaithi (2016). According to Stevenson et al. (2015), nurses need to reflect on their feelings after an incident, so they can discuss and share their emotional feelings with family or colleagues. Findings in this study show that (37.9%) of respondents reported that no action was taken against the perpetrator of physical violence by hospital management, which is similar to the findings of a study by Niu et al. (2019), which reported (33.5%) of participants indicated that no action was taken against perpetrators of violence. On a personal level, respondents indicated that in response to physical violence, they reported the incident (85.7%), and of those who reported the incident only 23.4% completed incident forms. Similar to this, a study by Banda et al. (2016) showed that nurses reacted to incidents of violence by reporting to a manager and completed incident forms.

In response to verbal abuse, current findings showed that verbal warning was issued against perpetrators, respondents reported the incident and completed incident form. Majority of respondents reported that they were not satisfied with the way hospital management handled incidents of violence. The current findings are consistent with other studies by Niu et al. (2019) and Alyaemni and Alhudaithi (2016).

### Strength and limitations

This study adds to the already existing body of knowledge which describes the occurrence of violence as experienced by nurses working in psychiatric settings. The population of the study included only nurses working in acute psychiatric units of three psychiatric hospitals in the province; findings cannot be generalised to other long-term psychiatric units and other psychiatric institutions in the country. The small number of respondents who reported an experience of violence makes it difficult to draw firm conclusions. Respondents had to answer recall questions, which would depend completely on their memory, and recall bias is possible.

### Recommendations

It is recommended that management should make efforts to come up with strategies that will reduce the incidence of violence towards nurses in their workplace. It is important to educate and encourage staff about importance of reporting incidents of violence and ensuring that the process of completing incident form is made simple and easy to follow. Support should be provided to staff who have experienced incidents of violence. This could impact staff performance of their duties, which will in turn improve the quality of patient care.

## Conclusion

The findings of this study are consistent with several studies, which alluded to violence against nurses in psychiatric units. The findings revealed that verbal abuse is the most reported incident by respondents, and female nurses reported experiencing maximum violent incidents. The significant association between years of employment as a nurse and the experience of physical violence by professional nurses is a concern, especially for nurses who are frontline workers in acute psychiatric units. It is important that psychiatric settings simplify the reporting process and encourage staff to report all types of violent and verbal abuse incidents, which will help in developing strategies in preventing and managing violent behaviours in the workplace. If a safe environment is to be created in psychiatric inpatients settings, violence perpetrated by patients has to be uncovered and be discussed openly. If that does not happen the well-being of staff and quality of care are compromised.
